# Prevention of extension lag using a sling attachment for Ligamentotaxor® devices in complex proximal interphalangeal joint injuries

**DOI:** 10.1308/003588412X13373405385214n

**Published:** 2012-07

**Authors:** S Gillespie, F Cashin, RJ Macfarlane, DJ Brown

**Affiliations:** Royal Liverpool and Broadgreen University Hospitals NHS Trust,UK

## BACKGROUND

Fracture subluxations at the proximal interphalangeal joint can be difficult to treat and variable in their outcome.^1,2^ A number of devices have been described that provide dynamic external fixation, allowing rehabilitation during the period of stabilisation.^3,4^ The Ligamentotaxor® device (Arex, Palaiseau, France) has been in use at our institution since 2008 and good results have been achieved. It was recognised that a small number of patients develop an extension lag at the distal interphalangeal joint while a Ligamentotaxor® device is in situ during treatment of fractures in the proximity of the proximal interphalangeal joint.

## TECHNIQUE

The sling attachment shown was devised in our unit. It is quick and simple to apply to the frame. It is manufactured from Velcro® and Orfit thermoplastic (Wijnegem, Belgium), and is easy to remove for exercise (if appropriate). It does not affect the normal functioning of the frame.

**Figure 1:**
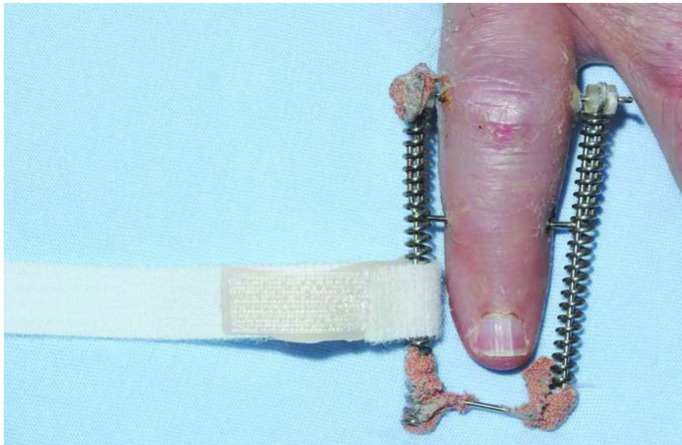
Velcro® is bonded to Orfit thermoplastic and attached to Ligamentotaxor® device

**Figure 2:**
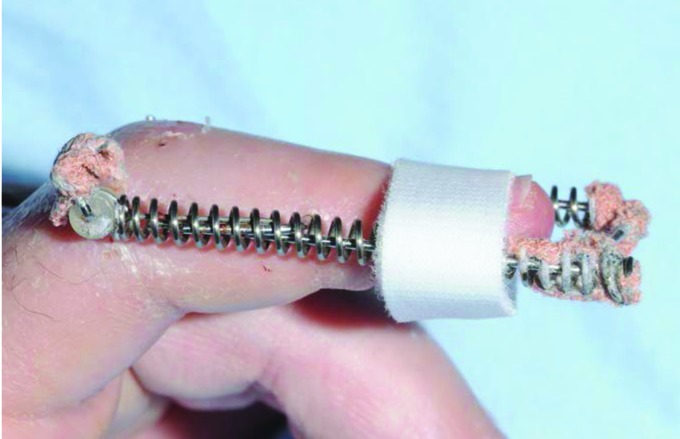
Sling is passed under distal phalanx, looped around device and fastened

1. Warmed Orfit thermoplastic is bonded onto ‘loop’ Velcro® approximately 2cm from one end.
2. The Velcro® strip is secured around one of the distal portions of the spring at the level of the distal phalanx (Fig 1).
3. The adhesive backed ‘hook’ Velcro® is adhered to the thermoplastic, passed under the distal phalanx and wrapped around the spring on the other side of the Ligamentotaxor® device, fastening to the hook Velcro® underneath, thereby supporting the distal phalanx (Fig 2).

## DISCUSSION

Prompt recognition of extensor lag and treatment using this frame modification arrests progression of the problem and facilitates its resolution. We recommend use of the Ligamentotaxor® sling in all cases complicated by extension lag.
